# Model-based genome-wide determination of RNA chain elongation rates in *Escherichia coli*

**DOI:** 10.1038/s41598-017-17408-9

**Published:** 2017-12-08

**Authors:** Peter Großmann, Anja Lück, Christoph Kaleta

**Affiliations:** 1 Research Group Theoretical Systems Biology, Friedrich-Schiller-University Jena, Ernst-Abbe-Platz 2, 07747 Jena, Germany; 2 Research Group Medical Systems Biology, c/o Transfusionsmedizin, Institut für Experimentelle Medizin, Christian-Albrechts-University Kiel, Michaelis-Straße 5, Haus 17, 24105 Kiel, Germany

## Abstract

Dynamics in the process of transcription are often simplified, yet they play an important role in transcript folding, translation into functional protein and DNA supercoiling. While the modulation of the speed of transcription of individual genes and its role in regulation and proper protein folding has been analyzed in depth, the functional relevance of differences in transcription speeds as well as the factors influencing it have not yet been determined on a genome-wide scale. Here we determined transcription speeds for the majority of *E. coli* genes based on experimental data. We find large differences in transcription speed between individual genes and a strong influence of both cellular location as well as the relative importance of genes for cellular function on transcription speeds. Investigating factors influencing transcription speeds we observe both codon composition as well as factors associated to DNA topology as most important factors influencing transcription speeds. Moreover, we show that differences in transcription speeds are sufficient to explain the timing of regulatory responses during environmental shifts and highlight the importance of the consideration of transcription speeds in the design of experiments measuring transcriptomic responses to perturbations.

## Introduction

Gene expression is a complex process, involving the timely interaction of many different proteins. Early on in the elucidation of this process it was noted that RNAP transcribes genes with different chain elongation rates (speeds)^1–4^, exemplified, for instance, by the two-fold difference in speed between the *trp* and *rrn* operons in *Escherichia coli* (17–20 nt/s^2^ and 41 nt/s^3^, respectively). High-throughput experiments recently uncovered transcription speeds varying between 5 and 60 nucleotides per second^5^ and single-gene-targeted experiments demonstrated gene- as well as growth-rate specific influences^6,7^.

The molecular causes for differences in transcription speed are unclear. As ribosomes bind to RNAP^8^ and the speeds of transcription and translation are linked^6^, control by tRNAs is possible. Gene sequence has an influence as well: *E. coli* rRNA operon gene sequences are transcribed with 65 nt/s and intergenic regions with 250–400 nt/s^9^. RNAP pausing sites depend on associated 10 nt hairpin structures^10^. Similar associations have been observed in eukaryotes. In *Drosophila melanogaster* exon-intron junctions are frequently transcribed slower than the remainder of the gene^11^ and in *Saccharomyces cerevisiae* exons are transcribed slower than introns^12^. A third factor influencing the transcription speed is the downstream DNA supercoiling level, the amount of twist and writhe of DNA: the accumulation of positive supercoiling slows down the transcribing RNAP^13^. Binding of DNA gyrase to specific sites and its ATP-dependent activity contravene excessive positive supercoiling^14–16^.

The speed differences seem to be necessary on a molecular level. Slow transcription at *S. cereviseae* exon-intron junctions is important for correct splicing^12^. Proper protein and RNA folding in *E. coli* can depend on a localized RNAP deceleration^9,17^. These mechanisms apply widely, but are insufficient to explain the large range of observed speeds, since the deceleration for protein folding happens only in some cases^18^ and RNA folding mostly applies to ncRNA or regions outside of genes^19,20^.

High transcription speed is costly for the cell. It increases the amount of supercoiling produced by transcription^21,22^, requires more ATP for DNA gyrase to remove positive supercoils^16^ and it increases the likelyhood of toxic R-loops upstream of RNAP which also limits gene evolvability^23^. However, high transcription speeds reduce the time until a functional product is produced and thereby enables a faster reaction to environmental cues.

Transcription speed has been inferred using various methods before: A short interval-time series of RNA-Seq after halting trancription initiation was analyzed for lags in RNA level drop on 482 genes in *E. coli* ^5^, and Global Run-On-Sequencing^24^ time series and ChIP-Seq were used to infer transcription speeds from transcription unit clearance waves after inhibition of transcription initiation in mouse embryonic stem cells^12^ and human breast cancer cell lines^25^. In addition, Fuchs *et al*.^26^ and Veloso *et al*.^27^ independently reported a method that also relies on a transcription stop and restart intervention before taking measurements. All of these approaches are limited to a minimum transcript length.

In this study, we infer transcription speeds on 2791 genes in *E. coli* from a collection of data sets integrated by an algebraic model without transcript length limits. We establish that the transcription speed reflects the relative importance of a gene and we identify the ontological groups subject to fast transcription and slow transcription for a defined growth condition. We propose an ecological purpose of different transcription speeds: The delay between the regulation of transcription initiation and a change of gene expression^6^ determines the reaction time of an organism to changes in its immediate environment. A case example of sequential down-regulation that can be largely explained with delays caused by different transcription speeds highlights the importance of considering the transcription speed in experimental setups.

## Methods

### Data Sets

We used *E. coli* K-12 MG1655 data sets whose experimental conditions were similar (Table 1). The growth conditions in Bernstein *et al*.^28^ differ from those in Cho *et al*.^29^ in temperature only and we assume the half life to be a relatively stable property of mRNA^28,30–33^.

**Table 1:** Data sets used for the calculation of genome-wide transcription speeds.

Data	Growth condition	Source
mRNA level	M9 minimal medium + 0.2 % glucose, exp. phase, OD _600_ = 0.6, 37 °C	Cho *et al*.^29^
RNAP ChIP-chip	M9 minimal medium + 0.2 % glucose, exp. phase, OD _600_ = 0.6, 37 °C	Cho *et al*.^29^
mRNA half life	M9 minimal medium + 0.2 % glucose, exp. phase, OD _600_ = 0.8, 30 °C	Bernstein *et al*.^28^

We extracted DNA sequence and genome annotations of *E. coli* K-12 MG1655 from NCBI GenBank record NC_000913.2^34^. CAI and tAI values for all genes were downloaded from HEG-DB^35^. A list of ncRNAs, a list of transcription factor proteins and their target genes, a list of essential genes in M9 minimal medium, a list of cytosol-located protein-coding genes and plasma membrane-located protein-coding genes featuring at least one transmembrane domain were extracted from the EcoCyc database version 19.5^36^. We obtained the operon structure from RegulonDB version 8.3^37^. We obtained gene-wise DNA gyrase density for M9 minimal medium from Jeong *et al*.^38^, protein concentrations in M9 minimal medium from Schmidt *et al*.^39^, transcription speeds in LB medium from Chen *et al*.^5^ and gene-wise H-NS density in LB medium from Kahramanoglou *et al*.^40^. We obtained dry mass amino acid concentrations in M9 minimal medium from Kaleta *et al*.^41^. We obtained the experimentally determined strength of the mRNA structure in MOPS medium from Burkhardt *et al*.^42^. We expect the mRNA structure to be mostly independent from growth conditions.

### Array Normalization

We first reproduced all data analysis as described in the original publications. The transcriptome tiling arrays were quantile normalized and the mean of perfect matches at all random probes was subtracted. We selected the larger value of forward and backward strand as RNA expression strength at each probe coordinate. The RNAP ChIP-chip tiling arrays were scaled to a median of 1, then quantile normalized and averaged at each probe coordinate. We excluded one of the three IP/mock-IP pairs because of strong selective differences to the other two. These differences are useful for finding transcription units, as was done by Cho *et al*.^29^, but would bias the RNAP density here. To correct for varying background and prevent correction-artefacts at long transcription units, we subtracted a running 10%-quantile window of size 30k nt from the averaged RNAP binding profiles. The rather large window size only marginally influences the corrected RNAP ChIP-chip signal at short transcription units compared to shorter window sizes.

### A Quantitative Model of Transcription Speed

The amount of RNAP bound to a gene and mRNA produced is generally assumed to be at an equilibrium in balanced growth^33,43^. The distribution of RNAP along the gene is not important in the state of equilibrium. Using the average amount of RNAP we can infer an average distance *d* between RNAPs along a gene by

1 $$d=\frac{l}{{n}_{p}},$$

where *l* is the length of a gene in nt and *n*_*p*_ is the number of RNA polymerases. We can express the average distance *d* also as the distance one RNA polymerase travels from the promoter until the next RNA polymerase inititates transcription:

2 $$d=\frac{v}{a},$$

where *v* is the average speed in nt/s and *a* is the promoter activity in 1/s, reflecting initiations/s. The promoter activity is cumulative for all promoters that initiate transcription for a certain gene. In this context it is not necessary to know which promoters are active. Solving for *v* we obtain

3 $$v=\frac{a\ast l}{{n}_{p}}$$

for stationary conditions. RNAP ChIP-chip experiments yield the density *d*_*p*_, a proxy to how many RNAPs are bound to a certain spot of DNA. *d*_*p*_ relates to *n*_*p*_ by

4 $${n}_{p}=\frac{l}{40\,{\rm{nt}}}{d}_{p},$$

with a 40 nt RNAP footprint in the elongation phase^44,45^, giving

5 $$v=\frac{a\ast 40\,{\rm{nt}}}{{d}_{p}}$$

in nt/s. We assume *d*_*p*_ background-corrected and scaled to express how much of a chosen stretch of DNA is physically covered by RNAP compared to how much RNAP could maximally fit onto it.

The promoter activity can be calculated under the steady state assumption. With that assumption the synthesis rate is equal to the degradation rate. At every time point the mRNA pool consists of mRNA from previous time points, including mRNA in the process of synthesis, and newly synthesized mRNA. The share of mRNA from previous time points is determined by the mRNA’s half life as equal to ${0.5}^{\frac{1}{\lambda }}$ according to the stationarity assumption. The mRNA level minus this share is the newly synthesized mRNA. Thus the promoter activity is

6 $$a={n}_{r}\ast (1-{0.5}^{\frac{1}{\lambda }})$$

in 1/s where *n*_*r*_, is the mRNA level and λ is the mRNA’s half life in seconds. The overall model for speed becomes

7 $$v=\frac{{n}_{r}\ast (1-{0.5}^{\frac{1}{\lambda }})\ast 40\,{\rm{nt}}}{{d}_{p}}\mathrm{.}$$

In this model linear measurement-related GC-bias in the mRNA level and RNAP density cancels as long as the RNAP density and mRNA level are measured on the same platform, e.g. the same type of tiling array. The model captures the degradation of mRNA in the process of synthesis^5^, as these mRNAs are usually measured along with free mRNA as the total mRNA level, *n*_*r*_. Transcription abortion within a gene affects both *n*_*r*_ and *d*_*p*_ in the same way and is hence implicitly corrected for in the model. Generally, physiological phenomena like collisions of RNAP and DNA polymerase are considered as far as their effect on transcription is fully reflected in the RNAP density, expression and half life data we use in our model.

Regulation delays, the lag between regulation at the promoter and an effective change in whole length transcript and protein level can be computed by

8 $$dela{y}_{k}={{\rm{\Sigma }}}_{i=1}^{k}\frac{{l}_{i}}{{v}_{i}},$$

where we add the delay of the preceding *k* − 1 genes to that of the *k*th gene in multi-gene operons. This analysis is only focused on intra-genic DNA.

### Speed Scaling

The data we use reflects absolute numbers of mRNA and RNAP density with an unknown linear scaling. Hence the model also returns transcription speed with an unknown scaling. We estimated this scaling by matching the speed at the reference gene *infB* (49.5 nt/s, interpolated from data in Vogel *et al*.^7^) at a typical growth rate of 60 min/doubling in this medium. While this work relies on the ranking of speed, the absolute speed provides the basis of our coarse-grained speed calculation validation. The RNAP footprint of 40 nt we assume in the model influences the outcome of this validation.

### Statistical Analysis and Software

All reported correlations are Spearman rank correlation coefficients with two-sided p-values. All reported p-values of comparisons between groups are calculated using the Mann-Whitney U-test. The p-values determined for amino acid and codon content are adjusted for multiple hypothesis-testing by the Benjamini-Hochberg method^46^ and were assumed significant for *p* ≤ 0.05. Only genes with non-zero transcription speed and background-corrected unscaled RNAP ChIP-chip signal >0.5 were analyzed. We did the Gene Set Enrichment Analysis (GSEA) using the PANTHER overrepresentation test^47,48^, release date 15. July 2016 with Bonferroni-corrected p-values and the Gene Ontology annotation with release date 30. November 2016. For each of the GO-classification trees Biological Process, Cellular Compartment and Molecular Function we removed unclassified genes and those with zero RNAP speed. We then used these as reference gene sets and for extracting the bottom and top 25% sets of transcription speed. We did the statistical analysis, modeling and data processing using the BioJava 3.0.4 library^49^ and R 3.4^50^ with the packages stat^50^ and psych^51^. We used bowtie 0.12.7^52^ with switches -a -S -trim5 3 -trim3 10 and the NCBI SRA toolkit^34^ for the reproduction of the RNA-seq read alignment in Chen *et al*.^5^. We used the R packages ggplot2^53^ and ggrepel^54^ for plotting and Biostrings^55^ as a convenient source of the universal genetic code table.

### Data Availability

All data generated or analysed during this study are included in the Supplementary Information.

## Results

### The Promoter Activity Equation is Robust Against Non-Stationarity

To check the robustness of our model against violations of the stationarity assumption, we validated it against experimental data from Zaslaver *et al*.^56^. The set contains promoter activities of 1920 *E. coli* promoters fused to *gfp* and shows little stationarity^57^. GFP fluorescence intensitiy and OD was measured in intervals of 14 min to a total of 54 time points both in M9 minimal and M9 rich medium. The promoter activity was calculated as dGFP/dt/OD^56^.

**Figure 1:**
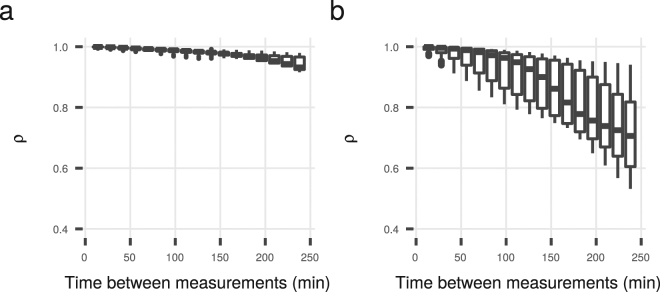
Pair-wise correlations of real promoter activities and our stationary state approximation over pairs of measurements with an increasing number of measurement points in-between in (**a**) M9 glucose minimal medium and (**b**) M9 rich medium. A part of the correlation drop in each medium can be explained by a decrease in cell size during the population increase^83^.

Though GFP in this experiment is stable, the *gfp*-mRNA is not. Megerle *et al*.^58^ have estimated the half life of this mRNA to be 6 min, making about 20% of the mRNA, and hence 20% of the increase in GFP, at each time step a leftover from the previous time step. We calculated two sets of promoter activities for all genes at each time point. Once we assumed stationarity and used equation (6) (reducing each intensity by 20%), and once we assumed non-stationarity and reduced each intensity by 20% of the previous time step’s intensity. Both sets of promoter activities correlated very well at each seperate measurement time point (*ρ* > 0.98) for both M9 rich and minimal medium. Even correcting every measurement with that of the 10th preceding measurement, corresponding to 154 minutes between measurements and simulating increasing fluctuations in the time series, the correlation coefficient between the real promoter activity and our approximation at each seperate time step was still >0.95 in minimal medium and >0.75 in rich medium (Fig. 1).

For completeness we mention here that the folding time of GFP of about 10 min is the rate limiting step in its photoactivation^59^, but since this delay applies to all steps of the time series equally, it does not have an effect on our calculations. We conclude that our calculation of promoter activity is very robust to violations of the stationarity assumption.

### Transcription Speed Distribution and Scale Validation

**Figure 2:**
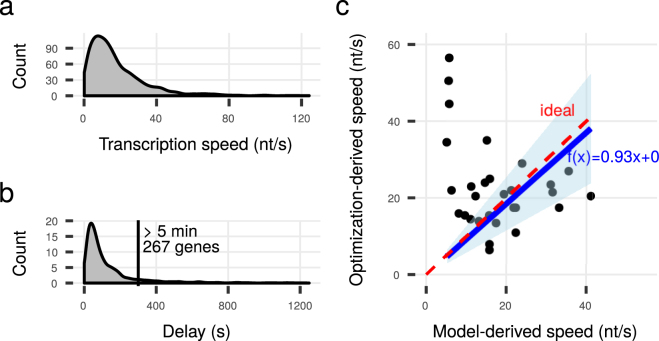
Transcription speed distribution and scale validation. (**a**) Transcription speed on 2791 genes in *E. coli*. (**b**) Time delay between transcription initiation and termination for genes with transcription speed >1. (**c**) Our model-derived transcription speed is on average close to the optimization-derived transcription speed. The shaded area marks the 95 % confidence interval.

Using equation (7) we obtained the transcription speed for 2791 genes (Fig. 2a). The distribution of transcription speed resembles the one reported by Chen *et al*.^5^. The mean speed is 17.86 nt/s (SD = 16.06). The genes with a determined non-zero speed in both the data of Chen *et al*. and our data have a mean speed of 24.59 nt/s (SD = 12.73) in the exponential phase on LB medium, 19.15 nt/s (SD = 13.29) in the stationary phase on LB medium and 24.48 nt/s (SD = 17.47) in the growth conditions of our data set.

To validate our scaling we computed the absolute amount of mRNA given a total of 1300 transcribing RNAP per cell^60^ and arrived at a sum of 6041 mRNA molecules (4876 mRNA molecules when we included operons with weak evidence). This is in the physiological range of 10^3^ to 10^4^ molecules, but closer to the approximately 8000 molecules expected for exponential growth on LB medium than the 3000 molcules expected in M9 minimal medium^61,62^. Assuming a total of 2400 mRNAs per cell^62^ we obtain 517 transcribing RNAPs (or 640 with weak-evidence operons). This is within the range reported by Stracy *et al*.^60^.

Hence, there is a tendency to an overestimation of transcription speed. However, the calculated transcription speed at rRNA-genes are below the expected value (44 nt/s, 65 nt/s expected). This is likely due to an overestimation of half lives as free rRNA in the original experiment did not decay through RNA maturation and incorporation into ribosomes^63^.

Next we fitted the clearance lag (the time till every RNAP has left a gene after initiation stops) as done in Chen *et al*.^5^ for each operon containing at least 3 genes to the time-series data for the mRNA half life^28^. Briefly, each time series shows a linear decay on a log-scale after some delay. The delay depends on the transcription speed, which we varied for each operon between 1 and 90 nt/s to find the one that gives optimal linear fits (maximum *R* ^2^) to the linear decay curves. In an ideal correspondence between the optimization-derived speed and our model-derived speed, a linear fit through them will have the slope = 1.00 and approximately so in the presence of measurement error. With a forced zero intercept we observed a linear fit with slope = 0.93 and its 95 % confidence interval encloses the ideal slope. The optimization-derived and our model-derived transcription speed were in good correspondence to each other (Fig. 2c).

### Central Transcriptional Regulator Proteins Have a Higher Transcription Speed

As a second, indirect approach to validate the determined transcription speed, we used them to assess the relative importance of genes in response to environmental challenges. We hypothesized that genes who’s transcription needs to be rapidly adjusted in response to an environmental cue, e.g. because they perform essential cellular functions, and those that are regulatory hubs in the gene regulatory network should have a faster transcription speed in order to minimize response times. To verify this hypothesis, we analyzed the transcription speed of transcription factor proteins relative to their number of target genes and genes essential for growth compared to non-essential genes.

**Figure 3:**
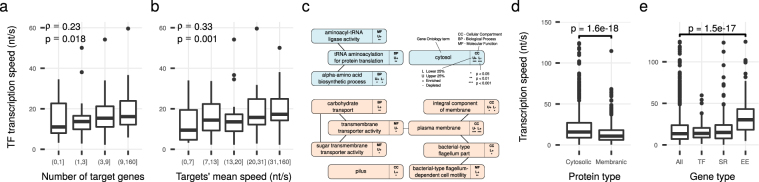
Association between transcription speed and cellular importance of genes. (**a**) Transcription Factor proteins (TFs) with a large number of target genes have a high transcription speed. (**b**) Transcription Factor proteins with a high transcription speed have targets with high mean transcription speed. (**c**) Selection of significantly enriched or depleted GO terms. Cytosol associated terms are in blue, membrane associated terms in red. Only terms at least 5 levels deep in the GO classification tree are displayed. The full results are in Supplementary Table T1. (**d**) Membranic proteins are transcribed more slowly than genes encoding exclusively cytosolic proteins. (**e**) The 113 essential genes (EE) in our growth condition are transcribed faster than average in contrast to stress response (SR) genes and Transcription Factor proteins.

We calculated regulation delays, that is, the time between the initiation of transcription and the completion of the transcript, for all genes (cumulatively for multi-gene operons) as the length divided by transcription speed. The regulation delay for most genes was <5 min, but 11% of genes had a delay of >5 min (Fig. 2b). We found only small differences in the delays between most transcription factor proteins (Supplementary Fig. S1). However, transcription speed was proportional to the number of target genes (*ρ* = 0.23, *p* = 0.018, Fig. 3a) and transcription speed of target genes (*ρ* = 0.33, *p* = 0.001, without self-regulation, Fig. 3b). Another way to shorten the regulation delay is to reduce the gene length. While we found that TF encoding genes are on average 12% shorter than all other genes, this difference is not significant (*p* = 0.234, data not shown).

Compared to all genes in the sample, essential genes are transcribed significantly faster (37.2 nt/s, vs. 17.9 nt/s, *p* = 1.5e − 16, Fig. 3e).

Low transcription delays enable a fast response to environmental stimuli, yet fast transcription is costly because of increased ATP demand and toxic R-loop formation. The similarity between transcription factor delays suggests that their transcription is fast enough, and an increase in speed is only present when strongly necessary.

### Cellular Localization Strongly Determines Transcription Speed

We analyzed functional enrichment as well as depletion of genes within the top and bottom 25% of the transcription speed distribution. These genes were mostly enriched or depleted for two distinct functional categories (Fig. 3c): synthesis of amino acids and the subsequent attachment to tRNAs, and localization to the plasma membrane and transmembrane transport. The cellular compartment “cytosol” is depleted in the bottom 25% (0.5-fold, *p* = 3.3e − 21) and enriched in the top 25% (1.5-fold, *p* = 4.7e − 16) and “plasma membrane” is enriched in the bottom 25% (1.5-fold, *p* = 8.7e − 11) and depleted in the top 25% (0.6-fold, *p* = 1.3e − 10) of transcription speed (Supplementary Table T1). Hence, membrane associated genes are transcribed slowly while cytosolic genes are transcribed quickly.

Plasma membrane proteins are slowly transcribed because of a structural coupling between transcription, translation and membrane insertion: To a large part, the plasma membrane contains helix-bundle membrane proteins. These proteins are mostly integrated into the plasma membrane via SecYEG and YidC during their synthesis^64^. RNAP is tied to the membrane and rotationally blocked by its spatial coupling to ribosomes^6,8^, and the ribosomes coupling to the membrane via the co-inserted nascent polypeptide chain. Therefore, the transcription-induced supercoiling is stronger around membrane-inserted genes^21^ and transcription speed should consequently be low to reduce the cost of the removal of supercoils. An example for this relationship is the increased supercoiling of a plasmid after replacement of the cytosolic gene with a membrane-bound gene of similar length and expression level^65^. Indeed, the mean transcription speed of inner membrane protein-coding genes (14.0 nt/s) is significantly lower than the mean speed of cytosol-targeted genes (20.4 nt/s, *p* = 1.6e − 18, Fig. 3e).

### Codon Composition and DNA Topology Co-Determine Transcription Speed

**Figure 4:**
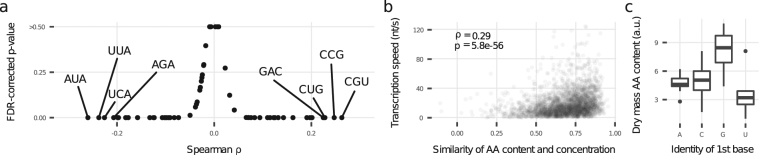
Codon distribution and amino acid (AA) availability impact transcription speed. (**a**) Relative frequency of codons vs. transcription speed. (**b**) Similarity of gene-wise relative AA content and AA concentrations correlate with transcription speed. Similarity is measured as Spearman correlation coefficient. (**c**) AA concentrations differ between codons’ 1st base identity (stop codons excluded).

Due to the coupling between transcription and translation, codons that strongly influence the speed of translation are also expected to influence the transcription speed. Indeed, relative codon frequencies are associated with transcription speed (Fig. 4a, Supplementary Table T2) as well as the bulk measures Codon Adaption Index (CAI, *ρ* = 0.39, *p* = 1.6e − 98, Supplementary Fig. S2) and tRNA Adaptation Index (tAI, *ρ* = 0.34, *p* = 9.8e − 76, Supplementary Fig. S2). Out of the 12 rare codons and the 2 codons with limited tRNA levels^17^, 13 significantly correlate negatively with transcription speed and 6 have the strongest observed negative correlations. Codons with a positive correlation often start or end with a C or G. The transcription speed positively relates to the GC content of the 1st base (*ρ* = 0.36, *p* = 4.4e − 86) and 3rd base (*ρ* = 0.22, *p* = 3.5e − 32), but not that of the 2nd base (*ρ* = 0.02, *p* = 0.4, Supplementary Fig. S2).

The 1st base typically has the strongest influence on the coded amino acid. To approximate cellular amino acid availability, we used amino acid abundance in cellular biomass as a reference^41^. We found that codons starting with G encode the most abundant amino acids (8.1 a.u., SD = 2.4, *p* = 0.002, Fig. 4c) and codons starting with U the amino acids with the lowest abundance (3.7 a.u., SD = 2.2, *p* = 0.003, Fig. 4c). Hence amino acid availability influences translation speed, probably by concentration-dependent aminoacyl-tRNA loading times at ribosomes.

The 3rd base-GC bias is probably influenced by decelerated translation through wobble-base pairing like it was observed in *Caenorhabditis elegans* and human HeLa cells^66^. Indeed, the frequency of strictly wobble-pairing codons influences transcription speed (*ρ* = −0.17, *p* = 1.3e − 19, Supplementary Fig. S4). This effect is even present when the codon has both wobble-pairing and non-wobble-pairing anticodons (*ρ* = −0.08, *p* = 9.9e − 06, Supplementary Fig. S4).

The secondary structure of mRNA has been associated with RNA pausing^10^ and strongly so with ribosome impediment^42,67^. Accordingly, transcription speed negatively correlates with the experimentally determined strength of the mRNA structure^42^ (*ρ* = −0.09, *p* = 6.4e − 03, Supplementary Fig. S4).

The correlation between CAI and mass spectrometry-derived protein levels (*ρ* = 0.58, *p* = 7.2e − 142, Supplementary Fig. S3) is not as strong between protein level and transcription speed (*ρ* = 0.19, *p* = 5.6e − 14, Supplementary Fig. S2).

Nucleoid associated proteins like H-NS have been suspected to act as a roadblock to RNAP^68,69^. We find no evidence of this effect when we compare the transcription speed in LB medium^5^ and H-NS density in LB medium^40^ (*ρ* = 0.01, *p* = 0.8, Supplementary Fig. S2). This is in support of the view that the binding strength of H-NS is low compared to the force with which RNAP traverses DNA^70^, although the binding strength can depend on the growth condition^71^. The binding of DNA gyrase on the other hand can have a positive effect on transcription speed. DNA gyrase is most active at certain sites along the genome^14,72^, but also binds with different strength gene-wise^38^ and binding positively influences the transcription speed (*ρ* = 0.23, *p* = 1.5e − 33, Supplementary Fig. S3). The likely cause for this association is the removal of otherwise elongation inhibiting positive DNA supercoils (speed reduction of 38–48%^13^).

### Transcription Speed-Derived Regulation Delays Explain Sequential Regulation

**Figure 5:**
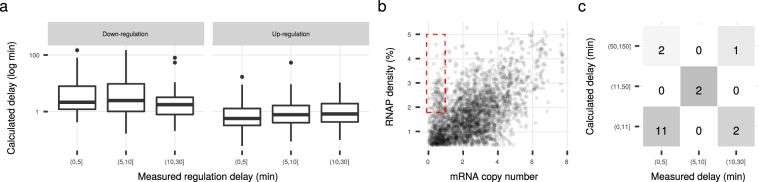
Calculated delays can explain measured regulation delays in stringent response. (**a**) Calculated delays are matching measured up-regulation delays better than down-regulation delays. Genes down-regulated first have the longest calculated delays. (**b**) Genes whose pool of mRNA has a large share of nascent mRNA (RNAP density >1.8 % and mRNA copy number <0.97). These genes seem to form a group in our data set and consist mainly of flagellar genes. (**c**) Confusion matrix for prediction of down-regulation interval from calculated regulation delay of flagellum genes. Numbers from bottom left to top right count correct predictions. The four wrongly predicted operons are *fliE*, *tsr* (top left), *fliFGHIJK* and *fliLMNOPQR* (bottom right).

Sequential regulation, i.e. ordered up- or down-regulation of multi-gene cellular systems or pathways, has been reported by Durfee *et al*.^73^ and differing regulation delays is one mechanism to explain this observation. They used three measurement time points up to 30 min post-intervention and in each one observed additional differentially regulated genes^73^. We find our calculated delays to predict the observed sequential regulation well in case of upregulation, but not in the case of down-regulation (Fig. 5a). Here the genes with the longest calculated delays are among the first down-regulated ones, implying the use of faster mechanisms that do not simply shut down transcription initiation but lead to faster mRNA inactivation, for instance through RNA-interference.

We then analyzed genes that have a high RNAP density and a low mRNA level, such that much of the measured mRNA level originates from nascent mRNA (see Fig. 5b). A gene set enrichment analysis of this group reveals a significant overrepresentation of genes for “bacterial-type flagellum-dependent swarming motility” (10.5-fold, *p* = 0.015). Durfee *et al*. report^73^ and we can substantiate a sequential down-regulation of flagellum genes as a consequence of regulation delays when the stringent response is invoked (Fig. 5c). However, our interval ranges only match those of Durfee *et al*. with regard to a scaling factor. There are two reasons for this. First, the microarray probes in Durfee *et al*.^73^ are at different positions in the genes, possibly at the start site in some cases, and they might indicate significant regulation before the gene is cleared of RNAP. Second, the mRNA half-lifes were determined on a time series of 8 minutes^28^, which is shorter than a portion of the regulation delays. The effect would be an overestimation of half-lifes and regulation delays, although we saw no such bias in the raw data of Bernstein *et al*.

### Random Forests Can Estimate Transcription Speed Without Using mRNA Half-Lives at Equal Growth Conditions

**Figure 6:**
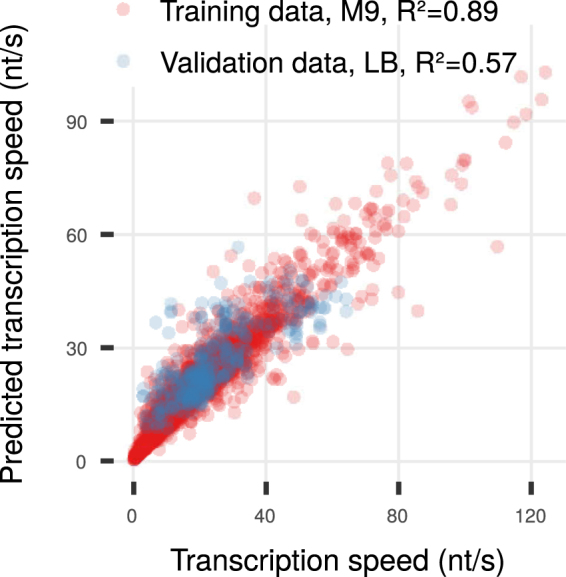
Prediction of transcription speed using a Random Forest not requiring mRNA half lifes. For the importance of each variable for prediction see Supplementary Table T3.

As measurements of mRNA half lifes require an elaborate experimental setup, we trained a Random Forest^74^, relying on better accessible measurements, to estimate the transcription speed. We trained it with default parameters and the variables CAI, tAI, 1st and 3rd base GC content, gene length, z-scored mRNA level, RNAP density and ratio of mRNA level to RNAP density. The Random Forest performed well on protein-coding genes in a 5-fold cross-validation scheme (${\bar{R}}^{2}=0.89$, *RRSE* = 0.32, Fig. 6). The most important variables were the mRNA level, RNAP density and their ratio by the internal variable importance ranking (see Supplementary Table T3). We then validated the trained Random Forest on transcription speed in LB medium^5^. The performance (${R}^{2}=0.57$, *RRSE* = 0.65, Fig. 6) indicates matched growth conditions as a requirement for the application of the trained Random Forest to other data sets. Training on a representative speed sample in the target condition might yield an acceptable performance.

## Discussion

The differences in transcription speed observed more than fourty years ago^1–4^, went largely unexplained and prompted an investigation into its causes and uses. Our approach is the first to shed light on transcription speed on a genome scale without limitations on gene length, including most of the active genes in *Escherichia coli* on minimal medium. Our model of transcription speed is robust against violations of the stationary state assumption and will be unreliable only when RNAP density is very low or when the transcription speed is very low. At low RNAP density, the measurement error will be amplified. At low transcription speed the regulation delay might become so large that it conflicts with the accurate determination of mRNA half-lifes.

Our model of the transcription speed includes the calculation of the steady state promoter activity. This promoter activity model for the first time enables a large scale inference and will thereby provide insights into factors that determine promoter strengths on a genome-wide scale. The analysis of the transcription speed set we obtained revealed that essential genes are transcribed very fast and that the transcription speed of transcription factor proteins that represent regulatory hubs is higher than those with a low number of regulatory targets. Many of the essential genes and regulatory hubs control resource intensive components of metabolism or maintenance. Low regulation delays help the fast adaptation of these molecular processes to changing conditions, but they require high speed transcription. High transcription speeds produce more positive DNA supercoiling that requires more ATP to be removed by DNA gyrase. In consequence, the optimal speed is achieved when this cost roughly matches the overall energetic benefit of a low regulation delay. Hence, a high speed implies a large benefit and thus high importance.

The high speed range of the speed distribution is functionally enriched with genes targeting the cytosol while the low speed range is enriched with genes targeting the cell membrane. This reflects *in vivo* on a genome scale the supercoiling-induced speed reduction found by Chong *et al*.^13^. The low transcription speed of membranic proteins is caused by the structural coupling of transcription, translation and transfer of the nascent peptide chain across the membrane. This structural coupling fully blocks the rotation of RNAP and causes the highest production of genomic supercoiling. Thus, compared to other genes the transcription speed of membrane-targeted proteins needs to be particularly low to avoid the potentially toxic side-effects of excessive supercoiling. This might be a reason for the expression of sugar transporters regardless of sugar presence, although the literature reports a high variability in expression of this group^39^ and only rare constitutive expression^75^. Because the structural coupling is so influential on transcription speed but effectively limited to a group of genes, the relative importance of gene products by transcription speed should be evaluated only within this group, but not between membranic proteins and cytosolic proteins.

The transcription speed is controlled largely by aminoacyl-tRNA concentrations in conjunction with the codon distributions and wobble-base pairing in *E. coli*, which we confirmed here genome wide. Wobble-base paring is known to slow down translation, and thus transcription, through a lower efficiency of aminoacyl-tRNA binding compared to non wobble-base pairing tRNA^66,76^. The aminoacyl-tRNA concentration is rate-limiting in translation^77^, hence the influence of the amino acid concentration in conjunction with the codon distribution. Transcription speed also has a negative feedback on itself through the transcription-coupled production of positive DNA supercoiling. Downstream positive supercoiling slows down transcription^13^. Positive supercoiling is released by the ATP-dependent enzyme DNA gyrase, which binds certain sites and prefers positively supercoiled DNA^78,79^. Thereby, the transcription speed not only depends on the DNA supercoiling level, but also on the presence of DNA gyrase binding sites and the energy state of the cell^79,80^. Our findings confirm this effect on a genome scale.

Our calculated transcription speed can be directly translated into the translation speed of the nascent transcript’s leading ribosome by virtue of the spatial coupling^8^. This limits the speed of the trailing ribosomes, but since ribosomes rarely queue^81^, translation is likely not faster than transcription in general. Translation dynamics, however, seem to dominate transcription dynamics, hence we can assume that translation speed matches transcription speed, as was shown before on a smaller scale^6^, even without a spatial coupling. Large scale experimental translation speed data in *E. coli* is to our knowledge not available. Large scale predictions of the translation speed are available^82^, but these are based on the codon distribution and tRNA copy number, which we already incorporated in our analysis.

We confirm on a genome-wide level that environmental conditions have a strong influence on the transcription speed. The speed scaling validation in Fig. 2 and the comparison of our data with that of Chen *et al*.^5^ indicates large variability between different media and growth conditions. Thus, to accurately determine the transcription speed, experimental data from matching growth conditions is required and a prediction of transcription speed across conditions is relatively inaccurate. Contributing factors likely are changes in DNA supercoiling level between conditions that strongly influences the transcription speed.

Transcription speed needs to be accounted for in time-series measurements with respect to time-series development and the timing of gene expression measurements after interventions. We observed delays as long as 20 min, much longer than the usual 5 min between intervention and first expression measurement. The majority of genes has a delay below 5 min, but accurate figures of expression change additionally depend on the transition time to post-intervention steady state. This transition time depends on the mRNA half life and the extent of changes in transcriptional activity. In the case of flagellum gene regulation during stringent response^73^, the regulation delays we obtained are sufficient to explain most of the step-wise down-regulation. They explain the regulation pattern qualitatively since the regulation delays are overall in the correct order but consistently higher than those observed in direct measurements.

Complementing our model with the appraoch of Chen *et al*.^5^ allows to calculate the RNAP density without ChIP techniques. Extending this combination by RNAP ChIP-Seq measurements is even better. It would yield accurate speed references for scaling, accurate low speed figures, absolute RNAP and mRNA numbers and more accurate high speed figures. The data from such an experiment could be used well to discern the complicated interactions of DNA supercoiling and transcription and the adjacent problem of topological domain formation.

## Conclusion

In summary, our work provides, for the first time, a genome-wide assessment of the mRNA-transcription speed, a method for determination without limits on transcript length based on experimental data sets and a comprehensive characterization of cellular factors influencing it. As we have shown, information on transcription speed can be used to determine the relative importance of genes for cellular function since such genes tend to be transcribed at faster speed as well as for the decision on the optimal timing of determining gene expression changes after a perturbation since the strength of effects strongly depends on the time it takes for transcription of affected genes to complete.

## Electronic supplementary material

Supplementary Figures and Tables

Supplementary Dataset 1
