# Supplementary material for: Model-based genome-wide determination of RNA chain elongation rates in Escherichia coli
Source: Sci Rep. 2017 Dec 8;7:17213. doi: 10.1038/s41598-017-17408-9 (PMC5722913; doi:10.1038/s41598-017-17408-9)
Supplement: Supplementary file 1 — Supplementary Figures and Tables [file 41598_2017_17408_MOESM1_ESM.pdf]

Model-based genome-wide determination of RNA chain elongation rates in *Escherichia coli*
Supporting Tables and Figures

Peter Großmann, Anja Lück, and Christoph Kaleta

July 13, 2017

List of Tables

1 Significantly enriched or depleted GO terms. 2
2 The contribution of different relative codon abundances to transcription speed. 6
3 Variable importance in speed prediction without mRNA half life 7

List of Figures

1 Essential genes have a short regulation delay while transcription factor proteins have an average regulation delay. 3
2 Genetic and dynamic properties that influence transcription speed. 4
3 Protein counts strongly correlate with the Codon Adaptation Index in M9 minimal medium. 4
4 Influence of codon wobbling and mRNA structure 5

Table 1: Significantly enriched or depleted GO terms.

	Reference list	Top 25%
Mapped IDs:	1746 out of 1746	429 out of 429
Unmapped IDs:	50	21
Multiple mapping information:	0	0

	Ref.	Top 25%				
GO biological process complete	#	#	expected	Fold Enrichment	+/-	P value
tRNA aminoacylation for protein translation	19	17	4.67	3.64	+	3.89E-03
↳tRNA aminoacylation	19	17	4.67	3.64	+	3.89E-03
↳amino acid activation	19	17	4.67	3.64	+	3.89E-03
↳cellular amino acid metabolic process	127	62	31.20	1.99	+	1.24E-04
↳primary metabolic process	813	243	199.76	1.22	+	9.85E-03
↳metabolic process	1054	313	258.97	1.21	+	1.71E-05
↳organonitrogen compound metabolic process	372	134	91.40	1.47	+	5.67E-04
↳organic substance metabolic process	977	290	240.05	1.21	+	2.98E-04
↳nitrogen compound metabolic process	655	200	160.94	1.24	+	3.96E-02
↳carboxylic acid metabolic process	240	95	58.97	1.61	+	8.58E-04
↳oxoacid metabolic process	243	96	59.71	1.61	+	8.08E-04
↳organic acid metabolic process	244	96	59.95	1.60	+	9.66E-04
↳small molecule metabolic process	407	142	100.00	1.42	+	1.41E-03
↳single-organism metabolic process	550	183	135.14	1.35	+	4.27E-04
↳cellular metabolic process	984	289	241.77	1.20	+	1.11E-03
↳organic substance biosynthetic process	506	175	124.33	1.41	+	6.21E-05
↳biosynthetic process	519	183	127.52	1.44	+	5.06E-06
↳cellular biosynthetic process	512	181	125.80	1.44	+	5.40E-06
↳cellular amide metabolic process	102	48	25.06	1.92	+	8.49E-03
↳organonitrogen compound biosynthetic process	271	111	66.59	1.67	+	1.31E-05
↳amide biosynthetic process	81	39	19.90	1.96	+	3.45E-02
sulfur compound metabolic process	63	34	15.48	2.20	+	1.16E-02
alpha-amino acid biosynthetic process	69	36	16.95	2.12	+	1.34E-02
↳cellular amino acid biosynthetic process	78	39	19.16	2.03	+	1.55E-02
↳carboxylic acid biosynthetic process	126	60	30.96	1.94	+	4.48E-04
↳organic acid biosynthetic process	126	60	30.96	1.94	+	4.48E-04
↳small molecule biosynthetic process	176	76	43.24	1.76	+	5.49E-04
↳single-organism biosynthetic process	317	118	77.89	1.51	+	6.88E-04

	Reference list	Bottom 25%
Mapped IDs:	1746 out of 1746	441 out of 441
Unmapped IDs:	50	8
Multiple mapping information:	0	0

	Ref.	Bottom 25%				
GO biological process complete	#	#	expected	Fold Enrichment	+/-	P value
bacterial-type flagellum-dependent cell motility	22	19	5.56	3.42	+	2.78E-03
↳archaeal or bacterial-type flagellum-dependent cell motility	22	19	5.56	3.42	+	2.78E-03
↳cilium or flagellum-dependent cell motility	22	19	5.56	3.42	+	2.78E-03
↳cell motility	27	21	6.82	3.08	+	4.35E-03
↳locomotion	37	29	9.35	3.10	+	7.06E-05
↳movement of cell or subcellular component	33	25	8.34	3.00	+	9.54E-04
↳cellular process	1275	285	322.04	.88	-	3.66E-02
↳localization of cell	27	21	6.82	3.08	+	4.35E-03
↳localization	316	115	79.81	1.44	+	1.13E-02
carbohydrate transport	42	27	10.61	2.55	+	7.17E-03
organic cyclic compound metabolic process	500	90	126.29	.71	-	2.76E-02
↳organic substance metabolic process	977	183	246.77	.74	-	4.17E-07
↳metabolic process	1054	200	266.22	.75	-	7.45E-08
cellular nitrogen compound metabolic process	542	97	136.90	.71	-	8.09E-03
↳nitrogen compound metabolic process	655	105	165.44	.63	-	3.13E-07
↳cellular metabolic process	984	186	248.54	.75	-	8.53E-07
alpha-amino acid biosynthetic process	69	3	17.43	< 0.2	-	1.22E-02
↳alpha-amino acid metabolic process	99	8	25.01	.32	-	2.90E-02
↳cellular amino acid metabolic process	127	9	32.08	.28	-	4.56E-04
↳primary metabolic process	813	155	205.35	.75	-	4.28E-04
↳organonitrogen compound metabolic process	372	39	93.96	.42	-	9.53E-10
↳small molecule metabolic process	407	59	102.80	.57	-	6.42E-05
↳single-organism metabolic process	550	100	138.92	.72	-	1.41E-02
↳cellular amino acid biosynthetic process	78	4	19.70	.20	-	8.82E-03
↳organonitrogen compound biosynthetic process	271	25	68.45	.37	-	6.69E-08
↳organic substance biosynthetic process	506	69	127.80	.54	-	2.44E-08
↳biosynthetic process	519	69	131.09	.53	-	2.71E-09
↳carboxylic acid biosynthetic process	126	13	31.82	.41	-	4.82E-02
↳organic acid biosynthetic process	126	13	31.82	.41	-	4.82E-02
↳small molecule biosynthetic process	176	16	44.45	.36	-	1.68E-04
↳single-organism biosynthetic process	317	36	80.07	.45	-	1.07E-06
↳cellular biosynthetic process	512	68	129.32	.53	-	3.93E-09
sulfur compound metabolic process	63	2	15.91	< 0.2	-	7.70E-03

	Reference list	Top 25%
Mapped IDs:	1727 out of 1727	424 out of 424
Unmapped IDs:	50	21
Multiple mapping information:	0	0

	Ref.	Top 25%				
GO molecular function complete	#	#	expected	Fold Enrichment	+/-	P value
aminoacyl-tRNA ligase activity	18	16	4.42	3.62	+	6.10E-03
↳ligase activity, forming aminoacyl-tRNA and related compounds	18	16	4.42	3.62	+	6.10E-03
↳ligase activity, forming carbon-oxygen bonds	18	16	4.42	3.62	+	6.10E-03
↳catalytic activity	1066	313	261.72	1.20	+	4.21E-05
anion binding	172	68	42.23	1.61	+	2.83E-02
↳ion binding	360	127	88.38	1.44	+	2.73E-03
transmembrane transporter activity	211	28	51.80	.54	-	4.37E-02
↳transporter activity	238	33	58.43	.56	-	3.74E-02

	Reference list	Bottom 25%
Mapped IDs:	1727 out of 1727	436 out of 436
Unmapped IDs:	50	8
Multiple mapping information:	0	0

	Ref.	Bottom 25%				
GO molecular function complete	#	#	expected	Fold Enrichment	+/-	P value
sugar transmembrane transporter activity	23	17	5.81	2.93	+	4.52E-02
↳carbohydrate transmembrane transporter activity	34	22	8.58	2.56	+	3.22E-02
↳carbohydrate transporter activity	34	22	8.58	2.56	+	3.22E-02
↳transporter activity	238	89	60.09	1.48	+	3.91E-02
↳transmembrane transporter activity	211	81	53.27	1.52	+	3.71E-02
catalytic activity	1066	215	269.12	.80	-	4.11E-05
identical protein binding	200	24	50.49	.48	-	4.61E-03
anion binding	172	20	43.42	.46	-	1.25E-02

	Reference list	Top 25%
Mapped IDs:	1596 out of 1596	391 out of 391
Unmapped IDs:	50	21
Multiple mapping information:	0	0

	Ref.	Top 25%				
GO cellular component complete	#	#	expected	Fold Enrichment	+/-	P value
cytosol	771	273	188.89	1.45	+	4.73E-16
↳cytoplasmic part	778	274	190.60	1.44	+	8.40E-16
↳intracellular part	906	296	221.96	1.33	+	3.79E-13
↳intracellular	939	299	230.04	1.30	+	1.35E-11
↳cytoplasm	854	289	209.22	1.38	+	6.90E-15
integral component of membrane	283	44	69.33	.63	-	2.18E-02
↳intrinsic component of membrane	302	49	73.99	.66	-	3.79E-02
↳membrane part	339	57	83.05	.69	-	4.07E-02
↳membrane	717	114	175.66	.65	-	9.20E-09
plasma membrane	579	78	141.85	.55	-	1.25E-10
↳cell periphery	632	89	154.83	.57	-	9.81E-11

	Reference list	Bottom 25%
Mapped IDs:	1596 out of 1596	403 out of 403
Unmapped IDs:	50	8
Multiple mapping information:	0	0

	Ref.	Bottom 25%				
GO cellular component complete	#	#	expected	Fold Enrichment	+/-	P value
bacterial-type flagellum part	9	9	2.27	3.96	+	4.49E-02
↳bacterial-type flagellum	10	10	2.53	3.96	+	2.25E-02
↳cell projection	22	21	5.56	3.78	+	2.63E-05
↳cell projection part	9	9	2.27	3.96	+	4.49E-02
pilus	12	11	3.03	3.63	+	2.36E-02
plasma membrane	579	216	146.20	1.48	+	8.69E-11
↳membrane	717	251	181.05	1.39	+	1.53E-10
↳cell periphery	632	233	159.58	1.46	+	9.78E-12
integral component of membrane	283	100	71.46	1.40	+	1.79E-02
cytosol	771	96	194.68	.49	-	3.32E-22
↳cytoplasmic part	778	97	196.45	.49	-	1.74E-22
↳intracellular part	906	133	228.77	.58	-	4.93E-20
↳intracellular	939	150	237.10	.63	-	1.59E-16
↳cytoplasm	854	119	215.64	.55	-	1.44E-20

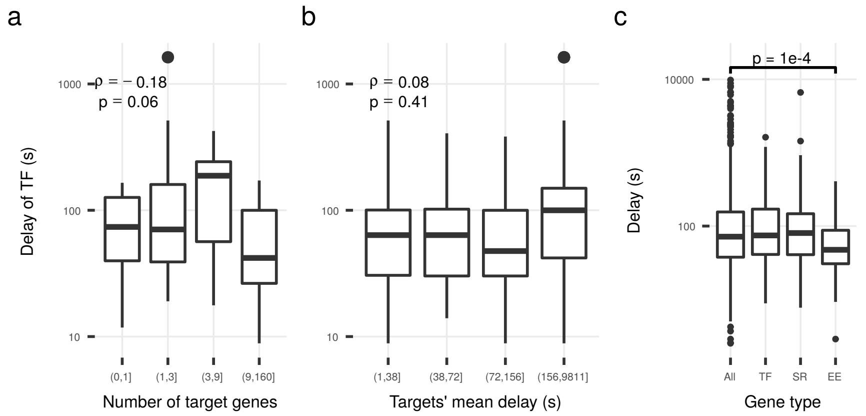

Figure 1: Essential genes (EE) have a short regulation delay while transcription factor proteins (TFs) have an average regulation delay. **a, b:** There is no general difference in regulation delays between TFs with different numbers of target genes or different mean regulation delays of target genes. **c:** The 113 essential genes in our growth condition have a lower than average regulation delay. The regulation delays of TFs and stress response (SR) genes overall do not differ from those of all genes.

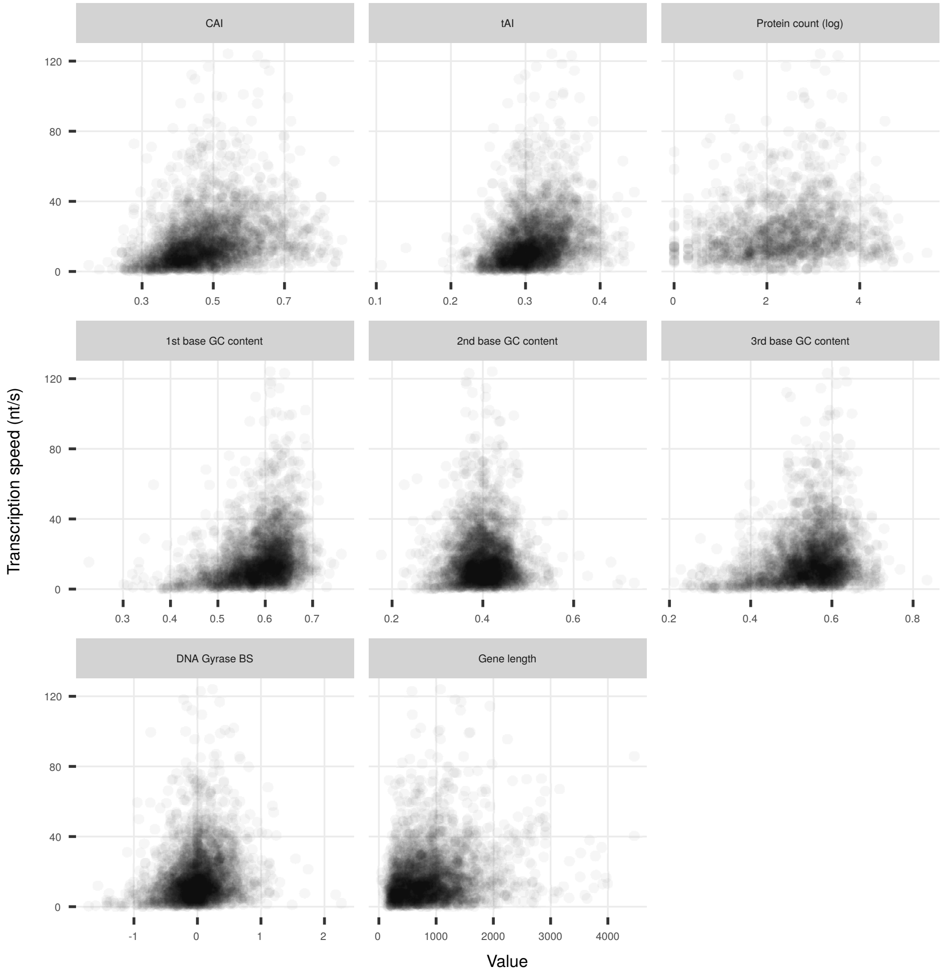

Figure 2: Each observation corresponds to one gene. DNA gyrase Binding Strength (BS) is measured in log-fold change in a two-colour microarray experiment upon cleavage at sites bound by DNA gyrase [2].

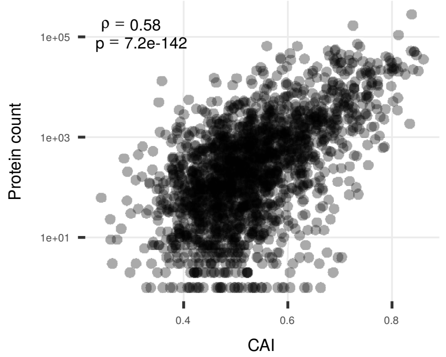

Figure 3: Protein counts strongly correlate with the Codon Adaptation Index in M9 minimal medium.

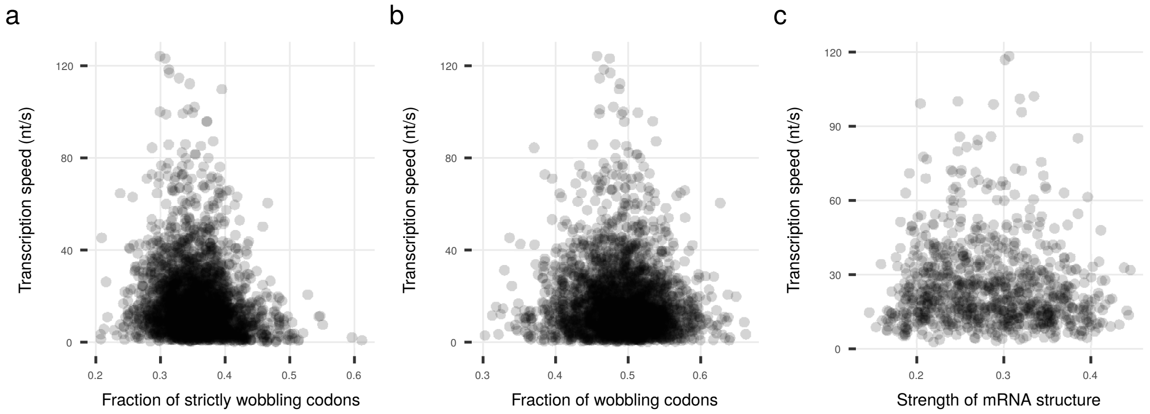

Figure 4: Codon wobbling and the strength of the mRNA structure negatively influence transcription speed. Codons with only **a:** wobble base paring tRNAs and **b:** those with additional non-wobble base pairing tRNAs slow down transcription. **c:** Strong mRNA structure tends to slow down transcription similarly to codon wobbling.

Table 2: The contribution of different relative codon abundances to transcription speed. a: correlation with transcription speed. b: belongs to the group of rare codons or has low associated tRNA levels [3]. c: has only strictly matching anticodons or has only wobble-base pairing anticodons [1].

Codon	ρ^a	p	Rare codon^b	Non-isoaccepted^c	Only isoaccepted^c
AUA	-0.26	2.220e-16	X		X
UUA	-0.237	2.220e-16	X	X	
UCA	-0.226	2.220e-16	X	X	
AGA	-0.207	2.220e-16	X	X	
AGG	-0.198	2.220e-16	X	X	
ACA	-0.197	2.220e-16	X	X	
AAU	-0.182	2.220e-16			X
UUU	-0.182	2.220e-16			X
GGA	-0.177	2.220e-16		X	
AGU	-0.158	2.220e-16			X
CGA	-0.158	2.220e-16			X
AUU	-0.133	7.124e-12			X
CUA	-0.125	1.107e-10	X	X	
CUU	-0.124	1.309e-10			X
UAU	-0.123	1.633e-10			X
UUG	-0.107	3.615e-08			
CCC	-0.102	1.565e-07	X	X	
CAA	-0.099	3.425e-07		X	
CGG	-0.099	3.425e-07	X	X	
UGU	-0.095	1.108e-06	X		X
UGG	-0.091	2.810e-06		X	
CCU	-0.082	2.439e-05	X		X
UAG	-0.073	1.718e-04			
GGG	-0.05	1.282e-02			
UGA	-0.039	5.827e-02		X	
ACG	-0.037	7.395e-02			
UAA	-0.035	8.631e-02			
AUG	-0.03	1.512e-01		X	
ACU	-0.029	1.665e-01			X
CCA	-0.026	2.005e-01	X	X	
UCG	-0.025	2.201e-01			
CUC	-0.024	2.356e-01		X	
CAU	-0.022	2.871e-01			X
GCU	-0.021	2.918e-01			
UCU	-0.017	3.958e-01			X
GUC	-0.009	6.730e-01	X	X	
GCA	-0.008	7.224e-01			
GUU	-0.007	7.334e-01			X
UGC	0.003	8.944e-01		X	
AAG	0.007	7.334e-01			X
GUA	0.009	6.730e-01			X
AAA	0.023	2.726e-01		X	
AGC	0.032	1.232e-01		X	
UCC	0.041	4.170e-02	X	X	
GCC	0.066	7.600e-04		X	
AUC	0.07	3.611e-04		X	
UAC	0.071	2.828e-04		X	
GAU	0.076	1.048e-04			X
UUC	0.083	1.998e-05		X	
GAG	0.094	1.310e-06			X
GUG	0.11	1.418e-08			X
CAG	0.115	2.870e-09		X	
AAC	0.122	2.253e-10		X	
CAC	0.125	1.130e-10		X	
GGU	0.132	9.286e-12			X
ACC	0.143	1.400e-13		X	
GCG	0.162	2.220e-16			X
GGC	0.191	2.220e-16		X	
CGC	0.197	2.220e-16			X
GAA	0.201	2.220e-16		X	
GAC	0.225	2.220e-16		X	
CUG	0.228	2.220e-16			
CCG	0.247	2.220e-16			
CGU	0.263	2.220e-16		X	

Table 3: Variable importance in speed prediction by Random Forest without use of the mRNA half life. The variables are ranked by importance, larger values indicate more important variables.

Variable	Importance
$\frac{\text{mRNA level}}{\text{RNAP density}}$	169.7
mRNA level	98.7
RNAP density	62.7
CAI	8.7
tAI	6.7
gene length	5.7
3rd base-GC bias	4.1
1st base-GC bias	3.5

References

[1] Hengjiang Dong, Lars Nilsson, and Charles G. Kurland. Co-variation of tRNA Abundance and Codon Usage in *Escherichia coli* at Different Growth Rates. *Journal of Molecular Biology*, 260(5):649 – 663, 1996.

[2] Kyeong Jeong, Jaeyong Ahn, and Arkady Khodursky. Spatial patterns of transcriptional activity in the chromosome of *Escherichia coli*. *Genome Biology*, 5(11):R86, 2004.

[3] Gong Zhang, Magdalena Hubalewska, and Zoya Ignatova. Transient ribosomal attenuation coordinates protein synthesis and co-translational folding. *Nature Structural & Molecular Biolology*, 16(3):274–280, 2009.
